# Classification of Breathing Phase and Path with In-Ear Microphones

**DOI:** 10.3390/s24206679

**Published:** 2024-10-17

**Authors:** Malahat H. K. Mehrban, Jérémie Voix, Rachel E. Bouserhal

**Affiliations:** 1 École de technologie supérieure, Université du Québec, Montréal, QC H3C 1K3, Canada; malahat.hajkarimi-mehrban.1@ens.etsmtl.ca (M.H.K.M.); jeremie.voix@etsmtl.ca (J.V.); 2 Centre for Interdisciplinary Research in Music Media and Technology (CIRMMT), Montreal, QC H3A 1E3, Canada

**Keywords:** breathing, in-ear audio, respiratory phases, hearables, breathing type

## Abstract

In recent years, the use of smart in-ear devices (hearables) for health monitoring has gained popularity. Previous research on in-ear breath monitoring with hearables uses signal processing techniques based on peak detection. Such techniques are greatly affected by movement artifacts and other challenging real-world conditions. In this study, we use an existing database of various breathing types captured using an in-ear microphone to classify breathing path and phase. Having a small dataset, we use XGBoost, a simple and fast classifier, to address three different classification challenges. We achieve an accuracy of 86.8% for a binary path classifier, 74.1% for a binary phase classifier, and 67.2% for a four-class path and phase classifier. Our path classifier outperforms existing algorithms in recall and F1, highlighting the reliability of our approach. This work demonstrates the feasibility of the use of hearables in continuous breath monitoring tasks with machine learning.

## 1. Introduction

Respiration is one of the most continuously monitored vital signs, which assists experts in detecting or predicting critical illnesses [1]. Several diseases such as asthma, bronchitis [2], chronic cough [3] and other pulmonary diseases involve the respiratory system, and can cause wheezing, sleep apnea [4], chest tightness, shortness of breath [5], and arrhythmia [6]. As the disease progresses, these symptoms become more severe [7]. For instance, chronic coughs are defined as coughing that continues for at least eight weeks [3] and acute bronchitis lasts more than three weeks [2]. Thus, long-term monitoring is required which implicates measuring under daily motion artifacts and real-world conditions. In addition to disrupting the patient’s daily life due to the associated symptoms, long-term monitoring using traditional methods would pose an unmanageable burden.

In primary care settings, generally, clinicians measure respiration by manually counting the number of breaths that patients take over a given time period. This process is not suitable for long-term monitoring, and is dependent on the operators who are experts in doing this task [8]. Sensors have been developed to measure the breathing rate of humans: Respiratory Inductance Plethysmography (RIP) measures chest movement caused by breathing [9,10], and is commonly used in medical and sports activity monitoring tools. However, this device, in addition to being expensive, large and uncomfortable for those who use it for a long period like while sleeping or in occupational settings, is also cumbersome, because RIP needs access to the entire chest and abdomen circumference to be able to do the measurement [11,12]. Monitoring respiration without disrupting the tasks that are being performed simultaneously would be beneficial. Also, it allows clinicians or even patients to recognize changes in breathing patterns over time which may cause early intervention, especially in progressive diseases such as chronic obstructive pulmonary disease (COPD) [13], for which early detection can be very helpful.

Existing contact-based methods, which include RIP, derivation of respiratory rate from electrocardiography (ECG) [14], or pulse oximetry, measure nasal airflow [15] or diaphragm movement [6]. However, they typically rely on specialized equipment and may require trained personnel to operate.

Within breathing analysis, the detection and classification of breathing path (nasal and oral) and breathing phase (inhalation and exhalation) are also of importance for diagnosis of a varied range of illnesses [16]. Mouth breathing is a voluntary and undesired way of breathing which affects the perioral muscles, tongue and cheeks [17]. Also, in [18], it was demonstrated that oral breathing can cause significant decreases in cognitive tasks, such as memory and learning capability. It is also a risk factor for dental health [18]. On the other hand, nose breathing, which is involuntary [19] and considered as “normal” breathing [20], may positively impact sleep quality, immunity, and body fat reduction [21]. More investigation in [18] shows nasal breathing causes more brain activation and connectivity compared to oral breathing.

Additionally, inhalation and exhalation, along with their respective characteristics, are vital for precise predictions and the effective management of infectious disease transmission. This importance arises from the fact that the exhaled air from infected individuals serves as a primary source of contagious viruses [22]. Additionally, monitoring the patterns of successive inhalation and exhalation can aid in anticipating the onset of neurodegenerative diseases, such as Parkinson’s disease [23].

Currently, wearable devices are increasingly being used to monitor vital signs [24], track body conditions like stress or fatigue level [25,26], and classify non-verbal events such as coughing or teeth clicking sounds [27]. Wearables could contain blood pressure sensors [28], accelerometers [29], and ECG and PPG sensors [30], which could track various health parameters such as heart rate, activity level, sleep, and respiration patterns. Among the various wearables equipped with different sensors and requiring placement on various parts of the body, in-ear wearables, or *hearables*, stand out for their ability to capture many signals while positioned inside or around the ear [27,31,32]. These devices can integrate a multitude of sensors, such as PPG, EEG, and ECG, enabling the monitoring of the wearer’s blood flow, brain, and heart activities [31]. Another way to track physiological signals with a hearable, is by using audio signals derived from non-contact [8,33] or contact microphones [34,35]. Generally, audio signals are an efficient method of tracking respiration [34,36]. These audio signals could be captured through microphones placed in mobile phones [33,37] and the recorded audio would be analyzed with phone applications to give information to the user. Despite the popularity and accessibility of this method, when the mobile phone is away from the user, monitoring and recording would be discontinued, requiring the mobile phone to always be nearby. Also, built-in microphones are more prone to record ambient noise which makes tracking respiration through audio somewhat inaccurate, and sounds may not be captured with sufficient clarity [38,39].

In addition to this method, physiological acoustic signals could be captured using the in-ear microphone (IEM) of a hearable [32,40]. Such a device was used in [32] to detect heartbeat and respiratory rate using traditional signal processing techniques such as envelope detection. This method relies on a proper acoustical seal between the earplug and the user’s ear canal to ensure a sufficient level for the breathing sounds [41]. Indeed, this acoustic seal attenuates the ambient sounds and, because of the occlusion effect, the low-frequency sounds of the wearer are amplified [42]. As a result, respiration sounds propagated to the ear canal through bone and tissue conduction are amplified and can be captured by the IEM for health monitoring applications [32].

***Contributions:*** To the best of our knowledge, this is the first work that classifies the phase and path of different types of breathing captured from an IEM. We present the parameters required for pre-processing the IEM signals to optimize the classification performance. We achieve approximately 87% accuracy when classifying breathing path. We present a 4-class classifier that detects both breathing path and phase with good performance. We benchmark our phase classifier against existing solutions, where it is shown to surpass the performance in recall and F1, demonstrating the reliability of our algorithm.

***Outline:*** The remainder of this paper is organized as follows: Section 2 describes the dataset, data pre-processing, feature engineering, and proposed ML algorithms. The results are provided in Section 3, followed by the discussions and conclusions in Section 4 and Section 5, respectively.

## 2. Methodology

### 2.1. Materials and Data Acquisition

**Figure 1:**
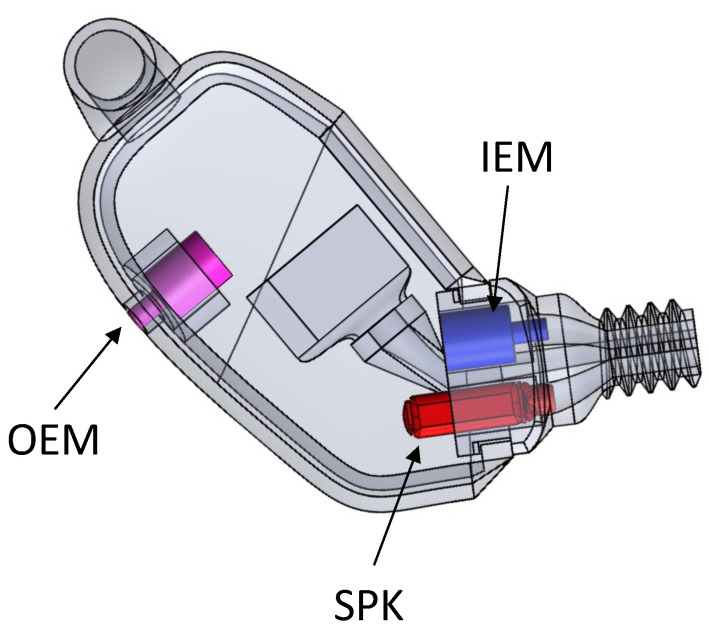
Illustration of the device worn by participants including an in-ear microphone (IEM), an outer-ear microphone (OEM), and a speaker (SPK).

**Figure 2:**
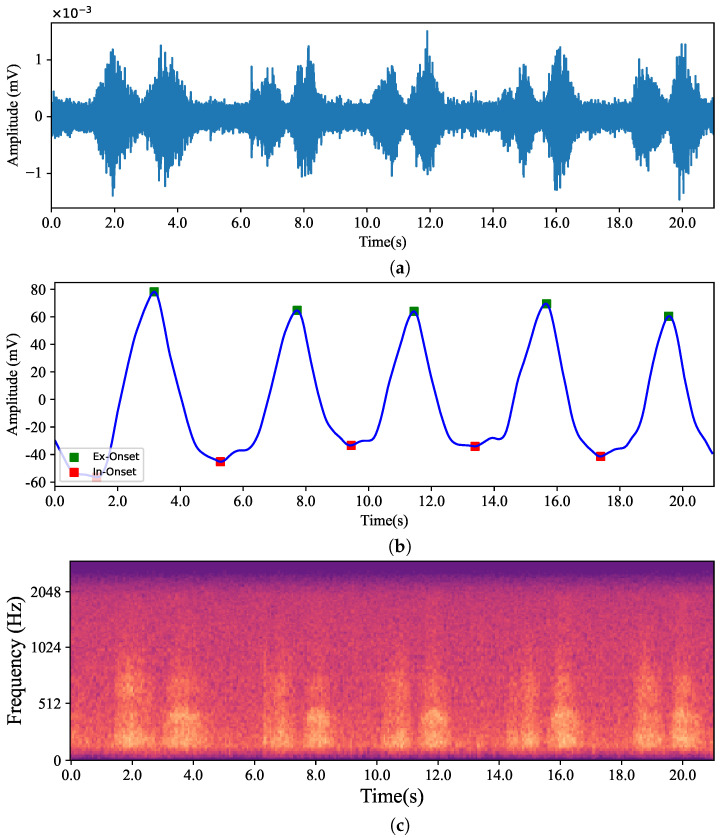
Respiration signal during normal nasal breathing captured simultaneously using an in-ear microphone (**a**) and the BioHarness 3.0 wearable chest belt (**b**). The mel-spectrogram of the in-ear microphone signal is presented in (**c**).

The data used in this work (approved by the *Comité d’éthique pour la recherche*, the internal review board of *École de technologie supérieure*) comes from an existing database of in-ear captured audio signal and body-captured physiological signals, iBad, as described in [32]. The database contains 160 recordings, which were captured from inside the left and right ear simultaneously using earpieces developed by the ÉTS-EERS Industrial Research Chair in In-Ear Technologies. The earpieces contained two microphones, one placed inside of the ear and one placed outside of the ear, as shown in Figure 1. The IEM enables the capture of audio signals through the occluded ear canal. The audio was recorded at a sampling rate of 48 kHz with 24-bit resolution. While recording audio from inside the ears, the BioHarness 3.0 wearable chest belt (Zephyr Technology Corporation, Annapolis, MD, USA) was used to capture ECG signals and respiration simultaneously to serve as a ground truth reference. Before each audio recording, the participants were instructed to breathe at different paces and intensities through their noses and mouths separately. As a result, the collected dataset contains recording that can represent the wide span of real-life breathing sounds. Table 1 summarizes the information related to the database, including the breathing types and their corresponding times, and abbreviations. A summary of the mean durations for inhales and exhales as well as the mean respiration rates for each breathing group are presented in Table 2. Some samples from the dataset were excluded due to the recordings being inaudible or the earpieces not being properly placed in the ear canal and failing to create an acoustical seal. Figure 2 presents a sample from the database of a participant breathing normally from the nose. It compares the respiration recordings obtained from the IEM and the chest belt, as well as a mel-spectrogram derived from the IEM signal.

**Figure 3:**
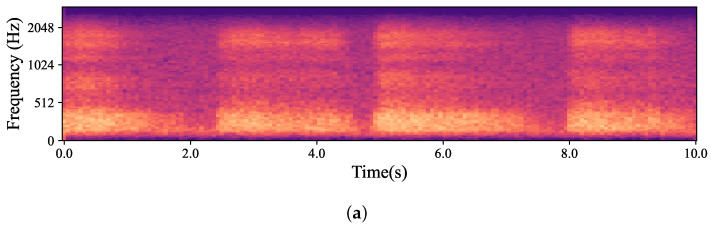

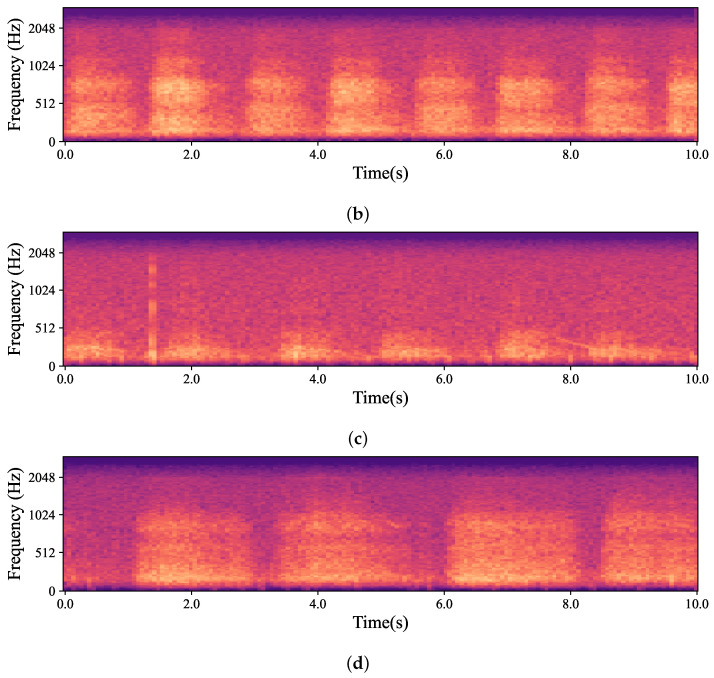
Mel-spectrogram obtained from the data captured by hearables. (**a**–**d**) show four randomly selected participants breathing normally through their noses after exercise. As depicted in the figures, each participant had a different breathing pattern, level and pace based on their physical fitness level and morphology. For example, in (**b**), the participant was breathing relatively fast and deeply while the participant in (**c**) had normal nasal breathing which was barely audible and distinguishable.

**Figure 4:**
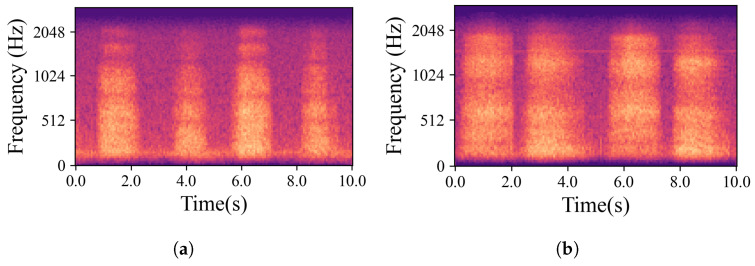

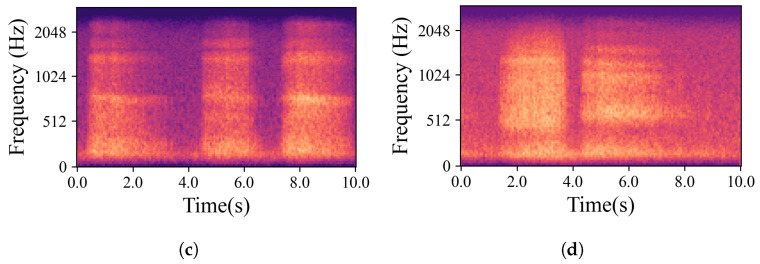
Examples of mel-spectrograms created from the IEM recordings. (**a**–**d**) illustrate breathing cycles, inhaling and exhaling, for four randomly chosen participants who were breathing deeply through their mouths. Individual differences did not significantly obscure the data; the recordings remained distinct and discernible.

The natural variations in ear canal shape between the right and left ear result in differences in fit, consequently yielding distinct audio signals from each ear. These signals represent the same physiological event captured simultaneously by both the left and right IEMs. For the purposes of this work, a sub-group of high-intensity signals, qualified as “*Forced*”, was created. The whole dataset is, hence, divided into two main groups based on the intrinsic intensity of the signals: namely *Forced*, as just described, and *All*, containing all the respiration signals. Signals labelled as *Forced* include nasal and oral fast and deep breathing, while signals labelled as *All* include *Forced*, normal nasal and oral breathing, as well as nasal and oral breathing after exercise. In Figure 3, which shows four normal nasal breathing after exercise, differences in the level of breathing among participants are observable. For example, in Figure 3c, the participant breathed calmly and steadily after exercise, making it barely audible and distinguishable, while others had higher intensity in their breathing. Conversely, in Figure 4, where participants were instructed to breathe deeply through their mouths, although there are still differences in breathing patterns between participants, the spectrograms show consistent amplitude and clarity across all recordings.

### 2.2. Pre-Processing

Pre-processing was performed to remove unrelated components to the breathing phase and path. Due to the bandwidth of bone and tissue conduction and the occlusion effect, an amplification of low and mid frequencies inside the occluded ear, no relevant information can be retrieved past 2 kHz [27,43]. To constrain the bandwidth of the relevant information, all signals were downsampled to 8 kHz. This choice, rather than downsampling to 4 kHz, was made to minimize the impact on the resolution of lower frequency components. The bandwidth of respiration signals recorded inside the ear typically falls between 150 and 2000 Hz. Therefore, all signals were filtered using a fifth-order Butterworth bandpass filter at those cutoff frequencies to remove any undesired noise. In the existing literature, which covers biosignal classification and detection, a 400 ms frame size with a 50% overlap was typically utilized to segment signals [27,44]. However, none of these prior works dealt with breathing signals in particular which led us to investigate this open research question by exploring what the optimal frame size for the breathing signal would be. The investigation involved comparing the frame sizes commonly found in the literature with those we empirically determined were the most effective for our purpose, as presented in Section 3. It should be noted that all the classifiers were trained and tested in two datasets with different segment lengths: once with 400 ms and 50% overlap which was chosen based on the literature [27,44], and once with 200 ms with 25% overlap after empirical testing.

### 2.3. Feature Extraction

In Section 2.1, how the fit level of each earpiece varies due to differences in the shape of the ear canals on the left and right sides was discussed. As a means of data augmentation, left and right recordings were considered separate signals thus duplicating the number of recordings [27].

Different time-domain and frequency-domain features were extracted from each recording. The time-domain features used were the Zero-Crossing Rate (ZCR) [33] and Root Mean Square (RMS) energy. Mel-frequency features simulate the auditory characteristics of the human ear and are widely used for the analysis of speech and acoustic breath signals [45]. Thus, the frequency-domain features extracted were the Mel-Frequency Cepstral Coefficients (MFCCs) and their derivatives (MFCCs delta and MFCCs delta delta), as well as spectral centroid (SC) and spectral roll-off (SR).

For each segment, 13 MFCCs, MFCCs delta and MFCCs delta delta were extracted, concatenated and considered as one feature named MFCC. Then, the MFCC feature vector was concatenated with the time-domain features to create the feature vector for each segment. This led to high-dimensional feature vectors (dimensionality of 604); therefore, Principal Components Analysis (PCA) [27,33] was used to reduce the number of variables in the feature space (reduced dimensionality of 35). Due to different measurement scales in the derived features, the feature vector was standardized to have a zero mean and unit standard deviation before forwarding it to the ML algorithm.

### 2.4. Machine Learning Classification Model

**Figure 5:**
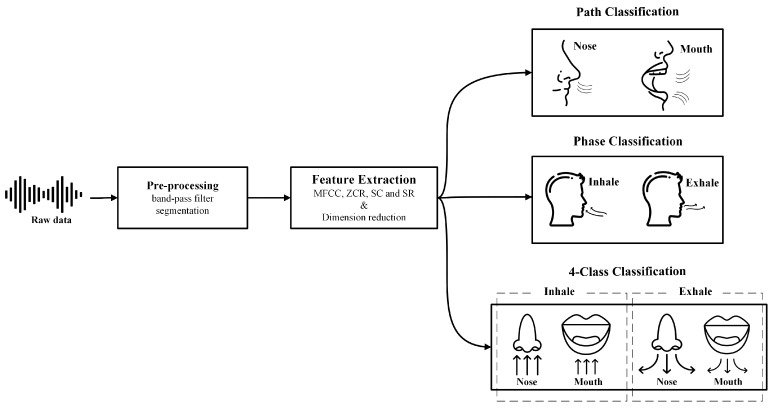
Proposed processing pipeline illustrating the three classifiers.

Three classification tasks were performed in this study. First, a binary classification of the breathing path into two classes, nose and mouth. Second, a binary classification of breathing phases, inhale and exhale. Third, a four-class classifier, combining the two binary classifiers. It was decided to merge the two first classifiers in order to determine if any enhancement in performance was possible. All three classifiers followed the same procedure. The same classifier was utilized to do the classification and compare the results. XGBoost was trained on the feature vectors derived from IEM signals as described in Section 2.3. To implement the algorithm, the Scikit-Learn library [46] in Python 3.2 language was used. To achieve the best performance, hyperparameters were optimized using Randomized Search Cross-Validation (RSCV). RSCV goes through a limited number of hyperparameter settings. It randomly moves within the grid to determine the optimal set of hyperparameters. As a result, unnecessary computations are reduced, and the tuned parameters are identified [46]. See Table 3, for the results of hyperparameter tuning carried out using RSCV. An overview of the proposed pipeline is presented in Figure 5.

### 2.5. Evaluation

Due to the limited amount of data, Cross-Validation (CV) was used to evaluate the performance of each classifier [46]. This method, also known as K-fold CV, divides the data into ‘K’ number of smaller groups to train the algorithm on ‘K-1’ groups and then test on one remaining group. In this work, the proposed classifiers’ performance was evaluated using a 5-fold CV. All classification models were divided into folds with equal distribution. Performance was evaluated using accuracy (ACC), precision (PR), recall (RE), and F1-Score.

## 3. Results

**Figure 6:**
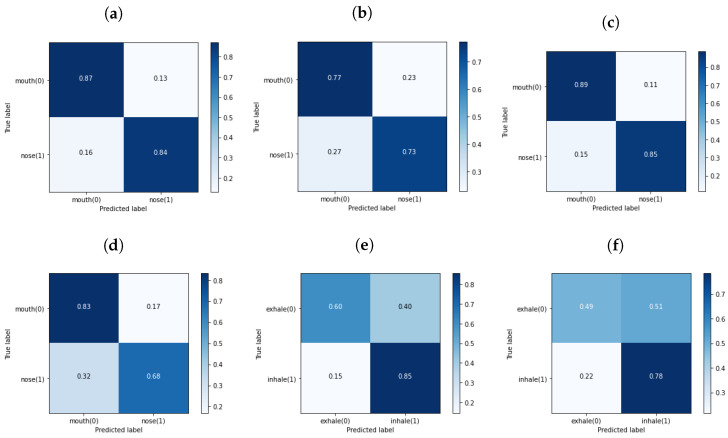
This figure depicts the mean CM values across all CV sets. (**a**) shows the results of *Nose*/*Mouth* classifier applied on *Forced* with the segment length of 400 ms, and (**b**) on *All*. The results of *Nose*/*Mouth* classifier applied on *Forced* and *All* with the segment duration of 200 ms are shown in (**c**,**d**), respectively. Finally, (**e**,**f**) represent the results of *Inhale*/*Exhale* classifier trained on *Forced* and *All* with the segment length of 200 ms, respectively.

Three classifiers were trained on samples of different lengths. Table 4 presents a summary of the number of samples used for each of the trained classifiers for every class. The initial classifier was trained to classify the breathing path, distinguishing between breaths originating from the nose and those from the mouth. The average confusion matrices (CM) of the breathing path classification across all CV folds with 400 ms segments are shown in Figure 6a for *Forced* and Figure 6b for *All*. The mean precision of the path classifier for *Forced* was 85.7% ± 0.4%, and *All* was 75.1% ± 0.2%. The rest of the evaluation parameters and their respective values are presented in Table 5. Further analysis to assess how the segments’ length would affect the classification was carried out with 200 ms segmentation. With a 200 ms frame length the mean precision of the classifier for *Forced* was 86.9% ± 0.2%, and 76.4% ± 0.1% for *All*. All evaluation parameters for the path classifier of 200 ms frames are presented in Table 6, and their corresponding confusion matrices are shown in Figure 6c,d. It is important to highlight that the average precision showed an increase of 1.1% for the *Forced* category and 1.2% for the *All* category when the segment length was reduced from 400 to 200 ms.

**Figure 7:**
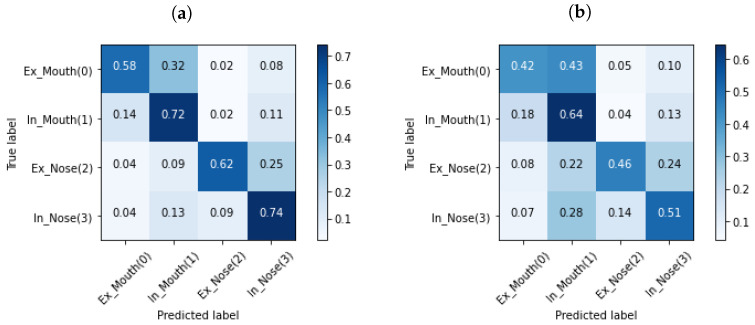
Mean confusion matrices showing four-class classifier results from using XGBoost and 200 ms segments. (**a**) shows the confusion matrix of *Forced* and (**b**) the confusion matrix of *All*. In both matrices, the confusing class was “Exhalation” showing that regardless of respiration path distinguishing exhalation from inhalation is complicated. Comparing (**a**,**b**), this gets worse when the algorithm is tested in *All*.

For both categories, *Forced* and *All*, the classifier’s performance was better for the 200 ms frames than with 400 ms frames. Thus, only the performance results of other classifiers are reported only for the 200 ms frames. For the phase classification, *Inhale*/*Exhale*, the average precision across all folds for *Forced* was 73.0% ± 0.5%, and for *All*, the average precision was 64.0% ± 0.1%. Table 7 and Figure 6e,f present all the evaluation parameters and corresponding CMs, respectively. As can be drawn from Figure 6e,f, the model performed effectively in identifying inhales, as evidenced by a high number of true positives. However, it struggled with the classification of exhales, with a notable presence of false positives and false negatives. Finally, the four-class classifier exhibited a mean precision of 68.7% ± 0.2% for *Forced* and 53.6% ± 0.1% for *All*. Table 8 and Figure 7 present the performance results of the four-class classifier.

## 4. Discussion

Only two studies that can be compared to our work were found in the literature [34,47]; however, both of these studies utilized data recorded by cellphone microphones. The abundance of data in these studies enabled the use of deep learning-based methods. The first study, named “Breeze” [34], focused on classifying breathing phases as a three-class classification: inhale–pause–exhale. In this study, participants were instructed to breathe at intervals of 4–2–4 s. In addition to instructing participants to breathe with specific timing intervals, they were asked to inhale through the nose and exhale through the mouth. Consequently, the data used in Breeze not only had defined timing, but also followed a specific sequence, enabling the algorithm to learn this temporal relationship and respiratory pattern [47]. The algorithm used in this work was convolutional recurrent neural network (CRNN), which yielded a precision of 69.02% [34].

In the second study, named “BreathTrack” [47], participants were not asked to breathe with a specific timing or pattern with a binary classification task: inhale or exhale. Convolutional neural network (CNN) was employed in this work, achieving a precision of 77.65% [47]. Additionally, BreathTrack utilized a dataset of 131 subjects, which was much larger compared to ours, allowing for a greater diversity of breathing patterns in the data. They were able to train the CNN with audio frames divided into 500 ms segments.

**Figure 8:**
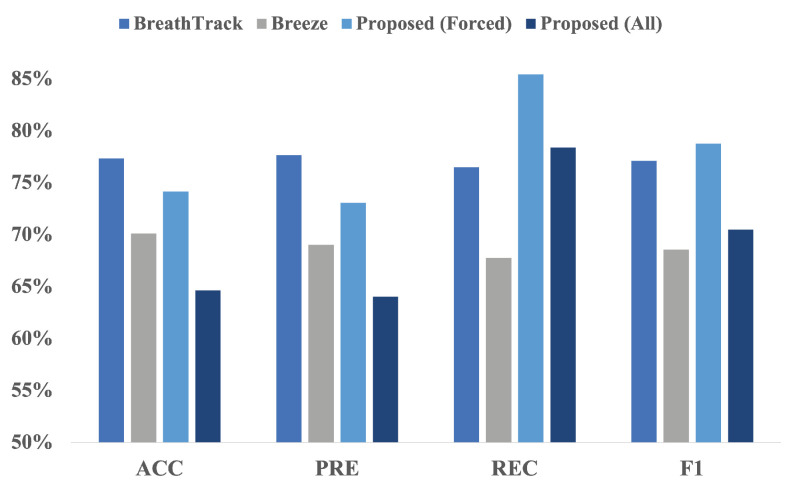
Comparison of ’BreathTrack’, ’Breeze’, and the proposed *Inhale*/*Exhale* classifier. Based on the figure, our proposed algorithm exhibits a higher recall and F1-score than the two other algorithms available in the literature.

In our dataset, participants could breathe at different intervals based on their breathing patterns, and even these time intervals could vary within each breathing cycle covering a more realistic range of possible breathing patterns. Figure 8 compares the performance of our algorithm for classifying breathing phases using data collected from IEM to the performance of “Breeze” and “BreathTrack”, the two algorithms for data collected from mobile phones. Looking at the bar chart, it seems that although our algorithm’s accuracy and precision may not be as high as the other two, its recall and F1-score are much better in both the *Forced* and *All* categories. This means our model is better at identifying actual positive instances, which is crucial for medical applications and achieving accurate predictions. Although extensive research has been conducted on breathing, to the authors’ knowledge, no other literature exists classifying *Nose*/*Mouth* breathing using audio signals.

When looking at the performance of classifiers, the best results belonged to the *Nose*/*Mouth* classifier. Although the classifier performed well along both *Forced* and *All* and both segmentation lengths, the highest outcomes were observed for *Forced* with 200 ms length. As *Forced* included recordings with fast and deep breathing, and all participants were instructed to breathe fast and deeply through both their nose and mouth, the data had a unified context. Thus, the algorithm successfully learned the context despite the individual variations in breathing patterns. On the other hand, in *All* alongside *Forced* items, other categories that were inherently challenging to identify were considered. In Figure 2c and Figure 3c, it can be seen that the normal breaths were so soft, and the distinguishing features had a level similar to the noise floor of the microphone. The recordings were also barely visible to the human eye in some cases in this category, which is obvious in Figure 3c. Additionally, normal breathing after exercise for both nose and mouth were dependent on the participant’s physical fitness level and inevitably influenced the outcomes. For instance, individuals who lead extremely sedentary lives, in comparison to well-trained people, are more likely to have deep breathing after doing exercise instead of breathing normally [48]. These differences are also observable in Figure 3. Also, given the *Inhale*/*Exhale* classifier results, it is clear that exhales were hard to classify. While breathing phase audio classification has shown higher accuracy in previous studies, it is important to emphasize the distinction between capturing breath sounds in front of the mouth versus within an occluded ear. Bone and tissue conduction act as a low pass filter, attenuating the acoustic features that differentiate an inhale from an exhale. The primary distinction lies in the exhale’s gradual rolling edge, where intensity and resolution steadily decrease, as opposed to the sharp edge of an inhale. In future work, it would be valuable to explore whether incorporating the OEM, as shown in Figure 1, could enhance classifier performance, given its bandwidth is more comparable to that of a cellphone microphone. As it is known that breathing through the nose and mouth results in unique sound patterns because of structural distinctions in the air passages [47], it can be interpreted that when the path of inhalation and exhalation is identified, the algorithm’s performance to classify the breathing phase increases. This performance improvement is observable in our four-class classifier. As shown in Figure 7, it can be concluded that knowing the breathing path beforehand improves the accuracy of classifying breathing phases, although the majority of errors still remain between distinguishing the exhale and the inhale. As an example, in Figure 7, the greatest error relates to misclassifying mouth exhalation (Ex-Mouth(0)) as mouth inhalation (In-Mouth(1)). Compared to the Forced category, this error is even higher when *All* is used.

The limitations of our work stem from the restricted number of participants and recordings. A limited number of recordings were conducted across a wide variety of breathing types, all using the same microphone. While this diversity provided a range of signals, it also resulted in fewer instances of each type, potentially making it harder to reinforce specific patterns for learning. Additionally, using the same microphone across all recordings limits the generalizability of our model, as the microphone’s frequency response, particularly in the low-frequency range, may have an important effect on the signal content. In addition, the quality of the recorded signals relied heavily on how well the hearable was placed in the ear canal. This resulted in participants having either a high occlusion effect, amplifying soft signals like normal breathing, or a low occlusion effect, which reduced the amplification of such signals. Consequently, this variability limited the performance of our classifiers, particularly in the *All* category. Despite these limitations, our findings provide valuable insights into breathing path and phase monitoring with hearables. Future endeavours will involve collecting more extensive datasets from a wide range of subjects and different types of microphones used in hearables, enabling the exploration of a larger group of breathing patterns in challenging conditions. This will allow us to employ both deep learning and unsupervised methods and compare algorithm performance.

## 5. Conclusions

We proposed a breathing phase and path classifier for breath sounds captured with an in-ear microphone that can achieve high accuracy using limited data. We reached optimal pre-processing parameters using a 200 ms window with 25% overlap. Using a simple and fast classical machine learning algorithm, XGBoost, trained on a small dataset, the breathing path classifier achieved an accuracy and recall of 86.8% when tested on clean data. An accuracy of 74.1% was achieved for the phase classifier with a recall of 85.4% under the same conditions. The results demonstrate the reliability of our proposed method in successfully classifying respiration path and phase. This suggests its potential application in long-term, real-life respiratory monitoring situations, offering a convenient solution for individuals who need to be observed continuously.

## Figures and Tables

**Table 1:** An overview of the dataset.

Groups	Abbreviation	Number of Recordings	Length (s)
Mouth breathing after exercise	BE	20	180
Normal mouth	BN	20	240
Deep mouth	BP	20	90
Fast mouth	BR	20	30
Nose breathing after exercise	NE	20	180
Normal nose	NN	20	240
Deep nose	NP	20	90
Fast nose	NR	20	30

**Table 2:** Mean durations of inhales, exhales, and overall respiration rates for each group.

Group	Inhale (s)	Exhale (s)	Respiration Rate (bpm)
BE	2.14 ± 0.8	2.1 ± 1.03	15.57 ± 4.15
BN	2.82 ± 1.16	2.31 ± 0.97	13.51 ± 4.91
BP	4.09 ± 1.5	3.4 ± 0.82	8.78 ± 3.3
BR	0.75 ± 0.45	0.78 ± 0.43	51.39 ± 25.42
NE	2.07 ± 0.78	2.16 ± 0.69	15.24 ± 3.47
NN	2.94 ± 1.12	2.61 ± 1.37	12.39 ± 4.04
NP	5.50 ± 2.96	3.96 ± 1.12	7.53 ± 3.13
NR	0.8 ± 0.45	0.8± 0.54	49.83 ± 24.90

**Table 3:** Hyperparameter tuning using RSCV. These results were derived from various combinations of the hyperparameters and evaluating the impacts of each combination on the algorithm’s performance.

XGBoost Hyperparameter	Description	Value
Learning rate	Regularization parameter. It shrinks feature weights in each boosting step.	0.1
max_depth	Maximum tree depth	6
min_child_weight	Minimum weight sum needed in a leaf node to stop partition.	1
subsample	Ratio of the training data sampled in each boosting iteration to grow the trees.	0.8

**Table 4:** Summary of the number of audio samples for each classifier and sample length.

Classifier	Data Group	Class	Samples	
			400 ms	200 ms
Phase	Forced	Exhale (0)	13,004	20,286
		Inhale (1)	17,004	25,874
	All	Exhale (0)	74,614	78,492
		Inhale (1)	86,548	91,695
Path	Forced	Mouth (0)		23,258
		Nose (1)		22,902
	All	Mouth (0)		90,487
		Nose (1)		79,700
4-class Path and Phase	Forced	mouth exhale (0)		10,538
		Mouth Inhale (1)		12,720
		Nose Exhale (2)		9748
		Nose Inhale (3)		13,154
	All	Mouth Exhale (0)		41,949
		Mouth Inhale (1)		48,538
		Nose Exhale (2)		36,543
		Nose Inhale (3)		43,157

**Table 5:** The mean and standard deviations of accuracy, precision, recall, and F1-score for the *Nose*/*Mouth* classifier were computed across all five folds using 400 ms data. Overall, when considering both *Forced* and *All* categories, the performance was superior in the *Forced* category.

	Evaluation Parameters			
Group	ACC (%)	PR (%)	RE (%)	F1 (%)
*Forced*	85.7 ± 0.4	85.7 ± 0.4	85.7 ± 0.4	85.6 ± 0.4
*All*	75.1 ± 0.2	75.1 ± 0.2	75.1 ± 0.2	75.1 ± 0.2

**Table 6:** The average and standard deviation values for accuracy, precision, recall, and F1-score of the *Nose*/*Mouth* classifier were computed across all five folds using 200 ms data. When assessing both *Forced* and *All* categories, the overall performance was better in the *Forced* category.

	Evaluation Parameters			
Group	ACC (%)	PR (%)	RE (%)	F1 (%)
*Forced*	86.8 ± 0.2	86.9 ± 0.2	86.8 ± 0.2	86.8 ± 0.2
*All*	76.1 ± 0.1	76.4 ± 0.1	75.7 ± 0.1	75.8 ± 0.1

**Table 7:** Comparison of *Inhale*/*Exhale* classifier performance on *Forced* and *All* categories. The 200 ms data were used for training and testing. Given all evaluation parameters in both *Forced* and *All*, the algorithm outperformed in *Forced*.

	Evaluation Parameters			
Group	ACC (%)	PR (%)	RE (%)	F1 (%)
*Forced*	74.1 ± 0.4	73.0 ± 0.5	85.4 ± 0.3	78.7 ± 0.2
*All*	64.6 ± 0.1	64.0 ± 0.1	78.3 ± 0.2	70.4 ± 0.1

**Table 8:** The mean ± standard deviation of the four-class classifier accuracy, precision, recall and F1-score across all five folds. The results were produced from 200 ms data. Across all evaluation parameters for both categories, the algorithm demonstrated better performance in *Forced*.

	Evaluation Parameters			
Group	ACC (%)	PR (%)	RE (%)	F1 (%)
*Forced*	67.2 ± 0.1	68.7 ± 0.2	66.4 ± 0.1	67.0 ± 0.1
*All*	51.4 ± 0.1	53.6 ± 0.1	50.7 ± 0.1	51.2 ± 0.1

## Data Availability

The original contributions presented in the study are included in the article, further inquiries can be directed to the corresponding author.
